# The cancer proteomic landscape and the HUPO Cancer Proteome Project

**DOI:** 10.1186/s12014-018-9180-6

**Published:** 2018-01-25

**Authors:** Connie R. Jimenez, Hui Zhang, Christopher R. Kinsinger, Edouard C. Nice

**Affiliations:** 1 OncoProteomics Laboratory, Department of Medical Oncology, Cancer Center Amsterdam, VU University Medical Center, Amsterdam, The Netherlands; 2 Department of Pathology, Mass Spectrometry Core Facility, Center for Biomarker Discovery and Translation, Johns Hopkins University, Baltimore, MD 21287 USA; 3 Office of the Clinical Proteomic, Tumor Analysis Consortium at the National Cancer Institute, National Institutes of Health, Bethesda, MD 20892 USA; 4 Department of Biochemistry and Molecular Biology, Monash University, Clayton, VIC 3800 Australia

**Keywords:** Human Cancer Proteome Project, International cancer proteomics initiatives, Clinical tumor proteomics

## Abstract

The Human Cancer Proteome Project (Cancer-HPP) is an international initiative organized by HUPO whose key objective is to decipher the human cancer proteome through a coordinated effort by cancer proteome researchers around the world. The ultimate goal is to map the entire human cancer proteome to disclose tumor biology and drive improved diagnostics, treatment and management of cancer. Here we report the progress in the cancer proteomics field to date, and discuss future proteomic developments that will be needed to optimally delineate cancer phenotypes and advance the molecular characterization of this significant disease that is one of the leading causes of death worldwide.

**Electronic supplementary material:**

The online version of this article (10.1186/s12014-018-9180-6) contains supplementary material, which is available to authorized users.

## Introduction

Cancer development is associated with deregulated signal transduction and aberrant protein activity and/or function as a result of genomic aberrations [1]. Current large-scale genomics efforts are fuelled by the hope that cancer genomic data may facilitate rational therapeutic decisions, guiding the selection of treatments tailored to the individual patient (personalized, precision or P4 (personalized, predictive, preventative, and participatory) medicine [2]. To date, cancer genomics has revealed hundreds of recurrently mutated or otherwise frequently aberrant genes, which may “drive” tumorigenesis [3, 4]. This effort has already led to numerous anticancer therapeutics that more precisely target cancer cells than do treatments that have been the mainstay of cancer care for decades, such as cytotoxic chemotherapy and radiotherapy. Currently there is a bias in the cancer research/oncology community toward using genetic testing, in particular DNA sequencing, to infer cancer biology and determine cancer patient management. However, we are still confronted with multiple challenges: (1) Only a small fraction of tumors harbor actionable mutations (~ 10%); (2) Resistance to treatment is frequent and invariably develops within a few months to a year; (3) The functional consequences of genomics alterations are often unknown or at best inferred; (4) Complicating matters further, clinical responses are tumor-context dependent. To overcome these challenges, complementary approaches in addition to genomics are needed to fully enable development of improved diagnostics and treatments as well as better informed individual patient care. To this end, comprehensive proteome analysis offers a means to measure the biochemical impact of cancer-related genomic abnormalities, including expression of variant proteins encoded by mutated genes, changed protein levels driven by altered DNA copy number, chromosomal amplification and deletion events, epigenetic regulation, and changes in microRNA expression [5]. Furthermore, analysis of post-translational protein modifications, in particular reversible protein phosphorylation, enables the detection of signaling network adaptations driven by genomic as well as micro-environmental changes [6].

To date, there are not many examples of cancer proteomics that have already resulted in improved routine care for patients as most discoveries are still in clinical development. One exception is the FDA-cleared multivariate index assay (IVDMIA) for OVA1 cleared by the FDA in 2009 (reviewed by Zhang and Chan [7], Cancer Epidemiology Biomarkers & Prevention 2010). The intended clinical use of OVA1 is to assess the likelihood an ovarian mass is malignant prior to a planned surgery. The clinical utility of OVA1 is to provide additional information for referral of patients with higher risk of malignancy to gynecologic oncologists. Ovarian cancer patients operated on by gynecologic oncologists are more likely to receive optimal cytoreductive surgery and treatment, and have been shown to have a better outcome. The discovery of biomarkers in the panel (other than CA125) using a proteomic approach and the development of the OVA1 IVDMIA algorithm played an important role in the design of the intended use for OVA1 and the clinical studies that led to the OVA1’s clearance by FDA [8, 9].

In addition, we would also like to highlight one recent study published in Annals of Internal Medicine [10] that has the potential to change clinical practice in the near future. In this very large-scale clinical cancer proteomics study in which 325 stool samples were profiled by label-free mass spectrometry, the authors report on new stool-based protein biomarkers for improved colorectal cancer screening. The identified markers yielded improved cancer detection over the gold standard hemoglobin marker and a five-fold higher detection rate of advanced adenomas [10]. As these lesions can be endoscopically removed, surgery then can be prevented. This has the potential to dramatically increase the impact of stool based colorectal cancer screening programs. Moreover, the new biomarkers can be detected with the same sampling method as used for the current fecal immunochemical (FIT) test. Upon further clinical validation, it will be easy to implement the new biomarker test in the FIT based screening programs.

This letter highlights current progress in applying high-resolution mass spectrometry-based proteomics approaches to bridge the gap between cancer genome information on the one hand and observed cancer phenotype on the other, as well as the steps forward required to unravel the cancer proteome, elucidate tumor-specific features, and identify protein targets for clinical application.

## Cancer proteomics and international collaboration to empower the precision medicine pipeline

Cancer is not a single disease, and each cancer type is heterogeneous. Therefore, to get useful insights into its pathogenesis, there is a need to profile in depth many tumors from individual patients and combine cancer proteomics data and genomic data sets for meta-analysis. In addition, big-data strategies that identify statistical associations are required to discover biological relationships. International collaboration is a vital component of this effort. A multidisciplinary approach is clearly needed [11], and to achieve progress, collaborative teams of researchers in the fields of cancer proteomics and genomics, computational biology, and bioinformatics are needed. Together, they can translate the enormous amounts of ever-increasing genomic and proteomic information into novel clinical knowledge and tools with a favorable impact for cancer patients around the world.

Key examples of outstanding international collaboration include the large, cancer-type specific studies carried out by the International Cancer Genome Consortium within the framework of The Cancer Genome Atlas project (TCGA), spearheading the description of the genomic landscapes of several thousands of tumors of over 20 tumor types [3, 4]. Moreover in the past 5 years, the Clinical Proteomic Tumor Analysis Consortium (CPTAC) of the National Cancer Institute has performed in-depth proteomic studies of genomically characterized tumors representing three major tumor types, i.e., colorectal, breast and ovarian cancer [5, 12]. These pioneering proteogenomic studies were recently published [13–15] and the data are freely available for analysis by the cancer proteomics community. Key insights were obtained on variant proteins arising from DNA/RNA variation, and on aberrant gene copy numbers and expression coupled to altered protein expression levels resulting from focally amplified chromosomal segments, pinpointing various cancer driver genes [13–15]. Another important observation made based on these three systematic cancer proteome studies was that proteome data outperform transcriptome data for coexpression-based gene function prediction [16].

Collaboration and data sharing are also key to the project. The Genomics Evidence Neoplasia Information, Exchange (GENIE) project is a recent endeavor of the American Association for Cancer Research (AACR) that aims to build an international cancer registry by sharing clinical cancer sequencing data from eight international institutions [17]. GENIE collects, catalogs, and links tumor genetic data with data on patient outcomes from all participating institutions and then makes the data publicly available. Finally, a new international initiative called APOLLO (Applied Proteogenomics OrganizationaL Learning and Outcomes) was launched as a Cancer Moonshot collaboration, that will utilize advanced genomic and proteomic expression platforms on high-quality human biospecimens in near real-time in order to identify potentially actionable therapeutic molecular targets, study the relationship of molecular findings to cancer treatment outcomes, and accelerate novel clinical trials with biomarkers of prognostic and predictive value [18]. The above efforts, together with on-going profiling studies in individual labs around the world, will hugely expand the description of cancer proteomes in the coming years.

## The Human Cancer Proteome Project

The framework for the Biology/Disease-driven Human Proteome Project (B/D-HPP) was established at HUPO 2010 in Sydney, Australia. Nine B/D-HPP workshops were then held at HUPO 2012 in Boston, at which it was decided to establish working groups focussing on specific biological processes and disease areas. Following these initial discussions, a number of additional working groups were established including the Ca-HPP which was co-chaired by the late Juan Pablo Albar (Centro Nacional de Biotecnología, Madrid, Spain) and Hui Zhang (Johns Hopkins University, Baltimore, USA) [19] and which is currently co-chaired by the authors of this letter.

By stimulating networking of cancer proteome scientists around the world and by organizing specific sessions at the annual HUPO meetings to share best practices and data, the Cancer-HPP aims to: (1) Delineate human cancer proteomes versus matched normal/premalignant samples; (2) Identify tumor-type specific signatures by comparison of multiple tumor types. (3) Develop standard operating procedures (SOPs) for the detection and measurement of these disease signatures. Importantly, it focuses on the analysis of human tumors, rather than experimental model systems, to help accelerate the translation of data into clinical practice.

To achieve our aims, we depend on the availability of high-quality specimens obtained according to strict SOPs to avoid protein degradation and minimize pre-analytical variability, while systematic clinical annotation is important for proper data analysis to correlate protein changes to clinical outcome. This will require multi-disciplinary collaboration between proteome scientists, (local) clinicians including pathologists. Pathologists are critical to the proteomics effort as they are in some cases collecting the sample, and in most cases processing and characterizing tissues, ensuring sufficient sample for clinical purposes, while also making significant efforts to bank as much tissue as is reasonable for research [20]. To underline specific recognition of this discipline in the cancer proteomics effort, recently a new pathology initiative proposed to support the HPP. Protocols for tissue collection and processing are available from the National Cancer Institute [21] as well as from links to partner labs on the Cancer-HPP website [22].

**Fig. 1:**
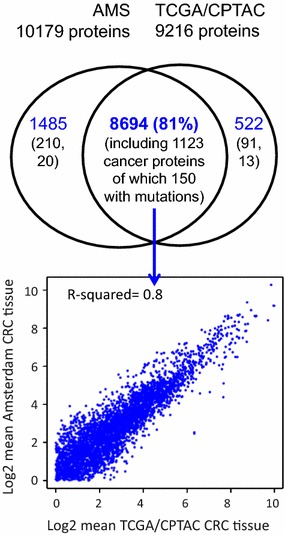
High overlap and correlated quantification of label-free shotgun proteomic data sets from two different laboratories. Venn diagram of overlap between colorectal cancer (CRC) tissue proteomics data sets produced in Amsterdam (AMS) by the Jimenez laboratory and in the USA by TCGA/CPTAC [13] (upper panel) and scatter plot of log2-transformed mean spectral counts for proteins in the overlap (lower panel). The proteome data were generated using different workflows and MS platforms (AMS: 5-band GeLC-MS/MS on a QExactive platform; TCGA/CPTAC: 12-fraction 2D-LC-MS/MS on an LTQ-Orbitrap), while for data analysis the same pipeline was used (MSFG + with ID Picker). The integrated CRC dataset contains 10,701 assembled proteins at 0.54% protein FDR and 0.1% peptide FDR. The result shows high inter-laboratory reproducibility of colorectal cancer proteomes generated for distinct sample sets, a prerequisite for successful meta-analysis and biomarker validation. Black numbers in the Venn diagram indicate annotation with a combined list of 2634 cancer genes/drivers from cancer genomics studies (Additional file 2: Table S2), revealing that 1123 proteins including 150 mutant cancer proteins were identified by both CRC proteomics studies

Furthermore, to ensure high-quality cancer proteome profiling and productive meta-analysis, we would like to emphasize the importance of assessing inter-laboratory reproducibility of workflow and intra-laboratory reproducibility with data collected in different time for data-dependent mass spectrometry (MS) for discovery, and the application of performance metrics to benchmark system performance on a regular basis using both simple and complex reference samples. Previous studies have confirmed the ability of targeted protein quantification by multiple reaction monitoring (MRM) to achieve reproducible, precise quantification of protein concentrations in tissues and biofluids across multiple laboratories throughout the CPTAC network [23]. Importantly, in our experience, when the whole proteomics workflow has been optimized and appropriate SOPs and reference materials are in place, there is also a good inter-laboratory and long-term correlation between label-free shotgun proteomics data that allow for relative protein quantitation, even when using different workflows (Fig. 1). Figure 1 shows an example of the overlap of identified proteins of the TCGA/CPTAC colorectal cancer proteome generated by 2D-LC–MS/MS of 95 tumors [13] and the proteome obtained for 40 colorectal tumors from patients around Amsterdam, generated by GeLC–MS/MS in the Jimenez laboratory. Not only does a large fraction of both proteomes overlap, but the spectral counts for the overlapping proteins are also highly correlated (Fig. 1). These encouraging inter-laboratory shotgun results indicate that meta-analysis of cancer proteomes is also possible in terms of relative quantitation when performing label-free proteomics. We expect that these results will further improve when using data-independent acquisition approaches.

**Fig. 2:**
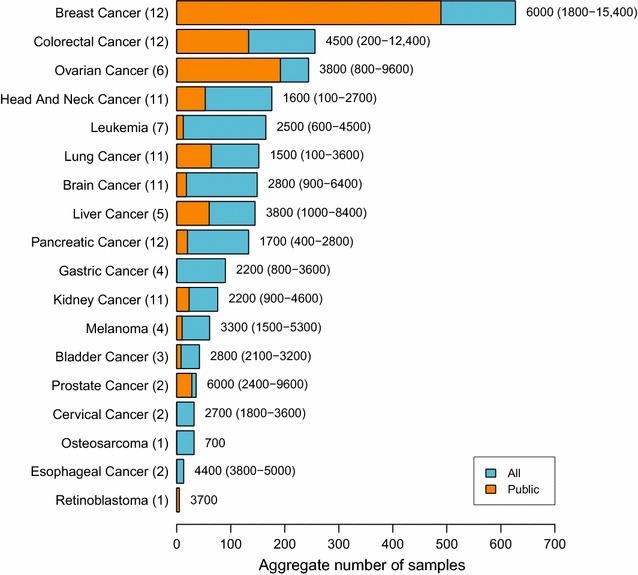
Aggregate sample sizes and average identified proteome sizes for high-resolution MS-based studies of cancer tissues. A meta-analysis of data sets reported in the literature for 18 different tumor types was performed. Per tumor type, the total number of samples analyzed was aggregated for all data sets (blue bars) or for publicly available data sets (overplotted orange bars). Next to the bars, the average number and range of identified proteins is shown. The number of data sets analyzed per tumor type is given in parentheses. The data on which this figure is based are provided in Additional file 1: Table S1

We strongly encourage all proteome scientists who report on human cancer proteomics studies to adhere to the clinical proteomics reporting guidelines as formulated in 2008 by an expert team for the Molecular and Cellular Proteomics journal [24] and the HPP Guidelines v2.1 (hupo.org/hpp/guidelines). This guideline mandates a protein-level FDR < 1%, careful scrutiny of the spectra, use of thresholds of 9 amino acids in length and 2 uniquely mapping peptides for peptide-to-protein matches, along with careful consideration of alternative protein matches, especially to sequence variants or isobaric PTMs of abundant proteins [25]. Finally, to achieve Cancer-HPP aims, post-publication deposition of raw data of cancer proteomics data sets in the public domain is crucial to enable meta- and pan-cancer analyses. Our recent literature survey revealed that raw data are only available for a quarter of the published cancer proteome studies (see below, Fig. 2). Therefore, this is an urgent call to make cancer proteome studies available in the public domain once they have been published.

## Cancer proteomes, where do we stand?

Three years ago, two international teams independently produced the first draft of the human proteome, largely using non-diseased human tissues and biofluids [26, 27]. Data sets were mainly collected from the public domain by the Kuster lab [26] and generated de novo by the Pandey lab [27]. These catalogs together represent ~ 80% of the human proteome (15,721 of 19,629 proteins; numbers reported on 27 July 2017 on the proteomicsdb website) that is available in a queriable database [28] and provides a baseline to better understand changes that occur in disease states. Upon the release of these two large-scale studies the authors received some criticism for overestimating the number of protein coding genes in their datasets [29]. The numbers currently reported on the proteomicsdb website contain the adjusted numbers.

To determine the current status of the human cancer proteome landscape, the Jimenez lab performed a PubMed analysis focusing on high-resolution mass spectrometry-based studies of the past 5 years, analyzing human cancers using the search terms tumor, human, cancer, proteome, proteomics, mass spectrometry in various combinations, together with dedicated searches using also the names of the tumor types (Fig. 2, Additional file 1: Table S1). This search returned hits for 18 tumor types and revealed that breast cancer proteome profiling stands out with the largest number of profiled samples (~ 600) in 12 different studies, followed by colorectal cancer and ovarian cancer studies with about 250 samples analyzed in 12 and 6 different studies, respectively (Fig. 2). For leukemias, lung, brain, liver, and pancreatic cancer, analyses were reported for more than 100 cancers, and for other tumor types, including gastric and kidney cancer, melanoma, bladder, prostate, and cervical cancer, osteosarcoma, esophageal cancer, and retinoblastoma, the numbers quickly drop. Therefore, this analysis highlights which tumor proteomes need to be profiled to have a comprehensive view of the cancer proteome. The analysis also revealed that many studies only analyzed 10–20 samples at a depth of 1000–2000 proteins per sample. Crucially, there is a need for studies with a more substantial numbers of samples (> 50), analyzed at a substantial depth (ideally > 3000–5000 proteins per sample). In addition, data deposition in the public domain will be key to allow for other researchers to re-use data and cross-validate biomarkers. Unfortunately, currently only a subset of the published tumor proteome data is publicly available (Fig. 2 and Additional file 1: Table S1). With MS instruments becoming ever faster and more sensitive, we expect that in the coming years the number of ‘better-powered’ studies, like those performed in the CPTAC context, will grow significantly. The results of the literature survey along with a meta-analysis of public domain and own data will be reported elsewhere.

Together, the world-wide cancer proteome profiling effort by the Cancer-HPP and allied initiatives will make it possible to build comprehensive and quantitative catalogs of proteins encountered in different tumor types and clinical conditions. Integration with functional genomics will address the basic question of how genotypic variability is mechanistically translated into phenotypic variability while integration with clinical data will enable application in a precision oncology pipeline.

## Outlook

The ability to interrogate cancer at the proteome level and integrate acquired knowledge with genome data will further improve clinical decision-making and catalyze new clinical and translational cancer research. The Cancer-HPP will support efforts that generate, analyze, and integrate cancer proteome data by disseminating best practices and aiming for development of a queryable data resource of published human cancer proteomes. We call upon all cancer proteome researchers, clinicians and pathologists to join us in this effort. Moreover, development of closer ties between proteome scientists and those that routinely develop, implement and oversee use of in vitro diagnostics (i.e. pathologists, clinicians, clinical chemists, IVD industry) is expected to provide another potential opportunity to accelerate progress in the cancer proteomics realm. We believe the future is bright, especially in view of the advent of novel mass spectrometry approaches that combine the best of discovery and targeted mass spectrometry and the development of emerging techniques like top down proteomics [30], which will facilitate the analysis of disease related post-translational modifications. Ultimately, cancer proteogenomics powered by precise measurements and high-quality diagnostic methods using the lowest “–plex” possible will realize the full potential of multi-parameter diagnostics and personalized medicine.

## Additional files

**Additional file 1: Table S1.** **Overview of high-resolution mass spectrometry-based studies of human cancers published in the past 5 years.** PubMed was searched with terms *tumor, human, cancer, proteome, proteomics,* and *mass spectrometry* in various combinations, including dedicated searches with the names of the various tumor types. Characteristic features of each study (aim, number of samples, MS platform, type of quantification, identification and validation of candidates, data download information, reference, and abstract) are detailed.

**Additional file 2: Table S2.** **Meta-analysis of human cancer genes.** Meta-analysis data on driver genes and mutated genes in cancer provided in eight publications were downloaded, mapped to official gene symbols, and gathered in a Combined List with gene-wise annotation of the number of studies implicating the gene. The file includes a list of kinase genes retrieved from the KinBase website maintained by the Manning lab (http://kinase.com/web/current/kinbase; original publication: Manning et al. The Protein Kinase Complement of the Human Genome. Science 2002; 298:1912-34), which was used to annotate kinase genes in the Combined List. References for the eight underlying publications are given in a separate tab.

## Abbreviations

2D-LC: two-dimensional liquid chromatography
AACR: American Association for Cancer Research
APOLLO: Applied Proteogenomics Organizational Learning and Outcomes
B/D-HPP: Biology/Disease-driven Human Proteome Project
Cancer-HPP: The Human Cancer Proteome Project
CPTAC: Clinical Proteomic Tumor Analysis Consortium
GeLC: gel electrophoresis followed by liquid chromatography
GENIE: Genomics Evidence Neoplasia Information Exchange
HUPO: Human Proteome Organization
MS: mass spectrometry
MS/MS: tandem mass spectrometry
SOP: standard operating procedure
TCGA: The Cancer Genome Atlas
